# Crystal structures of 3-chloro-2-nitro­benzoic acid with quinoline derivatives: 3-chloro-2-nitro­benzoic acid–5-nitro­quinoline (1/1), 3-chloro-2-nitro­benzoic acid–6-nitro­quinoline (1/1) and 8-hy­droxy­quinolinium 3-chloro-2-nitro­benzoate

**DOI:** 10.1107/S2056989019012799

**Published:** 2019-09-27

**Authors:** Kazuma Gotoh, Hiroyuki Ishida

**Affiliations:** a Department of Chemistry, Faculty of Science, Okayama University, Okayama 700-8530, Japan

**Keywords:** crystal structure, 3-chloro-2-nitro­bonzoic acid, 5-nitro­quinoline, 6-nitro­quinoline, 8-hy­droxy­qunoline, hydrogen bond

## Abstract

The structures of three compounds of 3-chloro-2-nitro­benzoic acid with 5-nitro­quinoline, (I), 6-nitro­quinoline, (II), and 8-hy­droxy­quinoline, (III), have been determined at 190 K. In each of the two isomeric compounds, (I) and (II), C_7_H_4_ClNO_4_·C_9_H_6_N_2_O_2_, the acid and base mol­ecules are held together by O—H⋯N and C—H⋯O hydrogen bonds. In compound (III), C_9_H_8_NO^+^·C_7_H_3_ClNO_4_^−^, an acid–base inter­action involving H-atom transfer occurs and the H atom is located at the N site of the base mol­ecule. In the crystal of (I), the hydrogen-bonded acid–base units are linked by C—H⋯O hydrogen bonds, forming a tape structure along the *b-*axis direction. Adjacent tapes, which are related by a twofold rotation axis, are linked by a third C—H⋯O hydrogen bond, forming wide ribbons parallel to the (03) plane. These ribbons are stacked *via* π–π inter­actions between the quinoline ring systems [centroid–centroid distances = 3.4935 (5)–3.7721 (6) Å], forming layers parallel to the *ab* plane. In the crystal of (II), the hydrogen-bonded acid–base units are also linked into a tape structure along the *b-*axis direction *via* C—H⋯O hydrogen bonds. Inversion-related tapes are linked by further C—H⋯O hydrogen bonds to form wide ribbons parallel to the (08) plane. The ribbons are linked by weak π–π inter­actions [centroid–centroid distances = 3.8016 (8)–3.9247 (9) Å], forming a three-dimensional structure. In the crystal of (III), the cations and the anions are alternately linked *via* N—H⋯O and O—H⋯O hydrogen bonds, forming a 2_1_ helix running along the *b-*axis direction. The cations and the anions are further stacked alternately in columns along the *a-*axis direction *via* π–π inter­actions [centroid–centroid distances = 3.8016 (8)–3.9247 (9) Å], and the mol­ecular chains are linked into layers parallel to the *ab* plane through these inter­actions.

## Abstract 2

The structures of the two isomeric hydrogen-bonded 1:1 co-crystals of 3-chloro-2-nitro­benzoic acid with 5-nitro­quinoline and 6-nitro­quinoline, and the 1:1 salt of 3-chloro-2-nitro­benzoic acid with 8-hy­droxy­qunoline have been determined at 190 K. In each crystal, the acid and base mol­ecules are linked by a short O—H⋯N or N—H⋯O hydrogen bond.

## Chemical context   

The hydrogen bonds formed between organic acids and organic bases vary from an O—H⋯N type to an O^−^⋯H—N^+^ type depending on the p*K*_a_ values of the acids and bases as well as inter­molecular inter­actions in the crystals, and at an appropriate Δp*K*_a_ [p*K*a(base) − p*K*a(acid)] value, a short strong hydrogen bond with a broad single minimum potential energy curve for the H atom or a double-minimum potential is observed (Schmidtmann & Wilson, 2008 ▸; Gilli & Gilli, 2009 ▸). For the system of quinoline–chloro- and nitro-substituted benzoic acids, we have shown that three compounds of quinoline with 3-chloro-2-nitro­benzoic acid, 4-chloro-2-nitro­benzoic acid and 5-chloro-2-nitro­benzoic acid, the Δp*K*_a_ values of which are 3.08, 2.93 and 3.04, respectively, have a short double-well O⋯H⋯N hydrogen bond between the carb­oxy O atom and the aromatic N atom (Gotoh & Ishida, 2009 ▸). Similar O⋯H⋯N hydrogen bonds have been also observed in compounds of phthalazine with 3-chloro-2-nitro­benzoic acid and 4-chloro-2-nitro­benzoic acid with Δp*K*_a_ values of 1.65 and 1.50, respectively (Gotoh & Ishida, 2011 ▸), and of iso­quinoline with 3-chloro-2-nitro­benzoic acid with Δp*K*_a_ = 3.58 (Gotoh & Ishida, 2015 ▸).
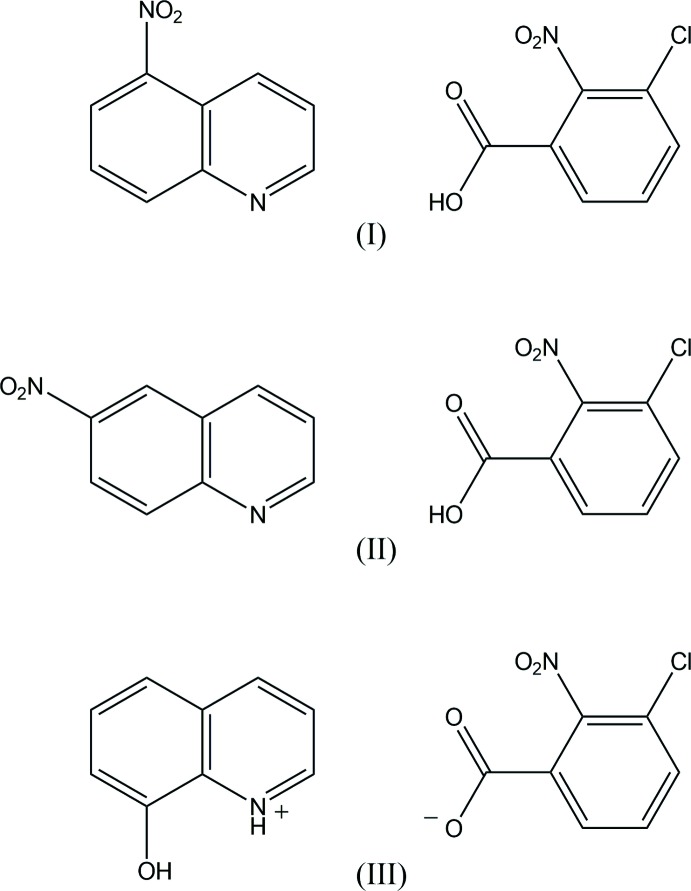


We report here the crystal structures of the title compounds in order to extend our studies of short hydrogen bonding in pyridine derivative–chloro- and nitro-substituted benzoic acid systems. The Δp*K*_a_ values are 0.98 and 1.42 and 3.02 for 3-chloro-2-nitro­benzoic acid–5-nitro­quinoline (1/1), (I), 3-chloro-2-nitro­benzoic acid–6-nitro­quinoline (1/1), (II), and 8-hy­droxy­quinolium 3-chloro-2-nitro­benzoate, (III), respectively.

## Structural commentary   

**Figure 1:**
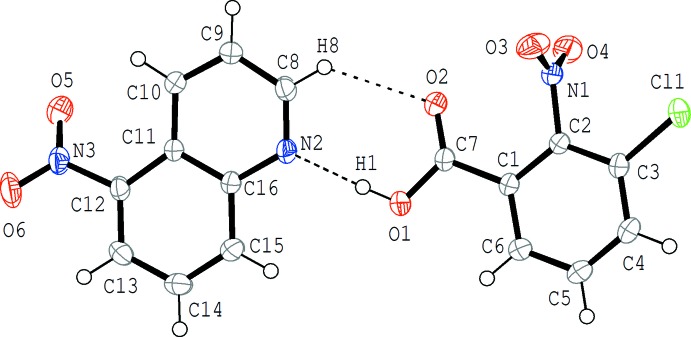
The mol­ecular structure of (I), showing the atom-numbering scheme. Displacement ellipsoids are drawn at the 50% probability level. The O—H⋯N and C—H⋯O hydrogen bonds are indicated by dashed lines (Table 1 ▸).

The mol­ecular structure of (I) is shown in Fig. 1 ▸. The acid and base mol­ecules are held together by an O—H⋯N hydrogen bond between the carb­oxy group and the N atom of the base. In addition, a weak C—H⋯O inter­action is formed between the acid and base mol­ecules (Table 1 ▸). In the hydrogen-bonded acid–base unit, the quinoline ring system (N2/C8–C16), the carb­oxy group (O1/C7/O2) and the benzene ring (C1–C6) are almost coplanar with each other; the carb­oxy group makes dihedral angles of 9.95 (12) and 9.45 (12)°, respectively, with the quinoline ring system and the benzene ring, and the dihedral angle between the quinoline ring system and the benzene ring is 2.59 (4)°. On the other hand, the benzene ring and the nitro group (O3/N1/O4) in the acid mol­ecule is almost perpendicular, with a dihedral angle of 86.14 (13)°. The quinoline ring system and the attached nitro group (O5/N3/O6) are somewhat twisted with a dihedral angle of 31.67 (11)°.

**Figure 2:**
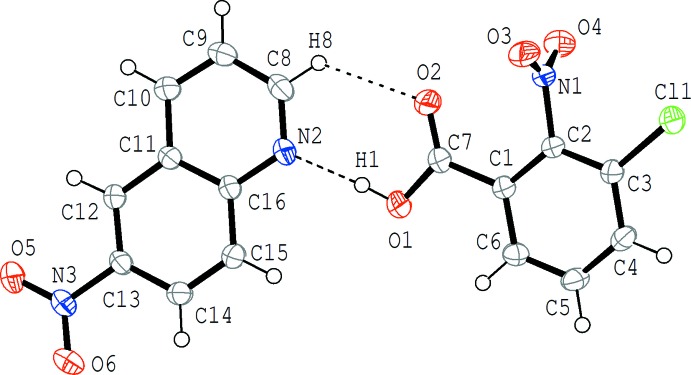
The mol­ecular structure of (II), showing the atom-numbering scheme. Displacement ellipsoids are drawn at the 50% probability level. The O—H⋯N and C—H⋯O hydrogen bonds are indicated by dashed lines (Table 2 ▸).

The mol­ecular structure of (II) is shown in Fig. 2 ▸. Similar to (I), the acid and base mol­ecules are held together by an O—H⋯N hydrogen bond and an additional C—H⋯O inter­action (Table 2 ▸). In the acid–base unit, the quinoline ring system, the carb­oxy group and the benzene ring of the acid are slightly twisted to each other; the carb­oxy group makes dihedral angles of 12.08 (13) and 2.40 (13)°, respectively, with the quinoline ring system and the benzene ring, and the dihedral angle between the quinoline ring system and the benzene ring is 10.99 (4)°. In the acid mol­ecule, the benzene ring and the nitro group (O3/N1/O4) are almost perpendicular with a dihedral angle of 88.54 (13)°. On the other hand, in the base mol­ecule the quinoline ring system and the nitro group (O5/N3/O6) are almost coplanar with a dihedral angle of 5.58 (12)°.

**Figure 3:**
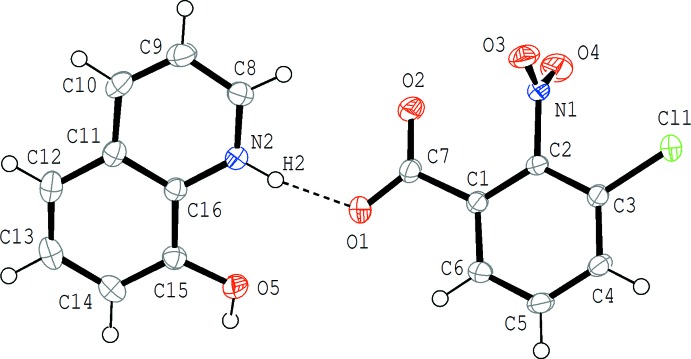
The mol­ecular structure of (III), showing the atom-numbering scheme. Displacement ellipsoids are drawn at the 50% probability level. The N—H⋯O hydrogen bond is indicated by a dashed line (Table 3 ▸).

The mol­ecular structure of (III) is shown in Fig. 3 ▸. An acid–base inter­action involving H-atom transfer occurs and the acid and base mol­ecules are linked by an N^+^—H⋯O^−^ hydrogen bond (Table 3 ▸). In the hydrogen-bonded unit, the quinoline ring system makes dihedral angles of 34.96 (13) and 30.80 (14)°, respectively, with the carboxyl­ate group and the benzene ring of the acid. In the acid mol­ecule, the benzene ring makes dihedral angles of 4.71 (13) and 86.12 (11)°, respectively, with the carboxyl­ate and nitro groups.

## Supra­molecular features   

**Figure 4:**
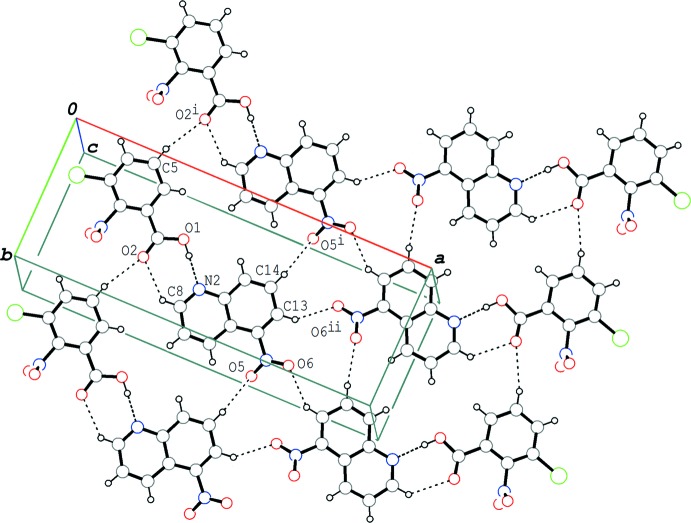
A packing diagram of (I), showing the hydrogen-bonded tape structure along the *b*-axis direction. Adjacent tapes, related by a twofold rotation axis, are linked by further C—H⋯O hydrogen bonds, forming wide ribbons parallel to (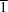
03). The dashed lines indicate the O—H⋯N and C—H⋯O hydrogen bonds. [Symmetry codes: (i) *x*, *y* − 1, *z*; (ii) −*x* + 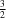, *y* − 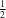, −*z* + 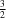.]

**Figure 5:**
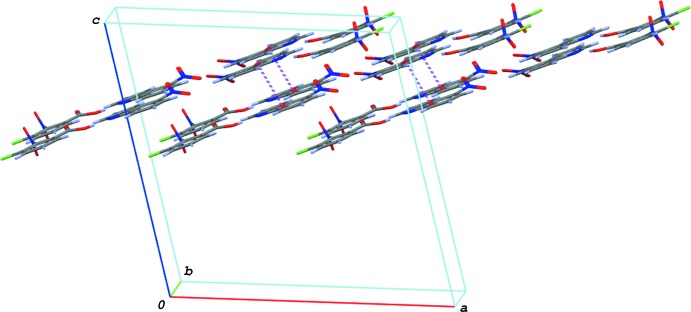
A partial packing diagram of (I), showing the ribbons linked by π–π stacking inter­actions (magenta dashed lines).

In the crystal of (I), the hydrogen-bonded acid–base units are linked by C—H⋯O hydrogen bonds (C5—H5⋯O2^i^ and C14—H14⋯O5^i^; symmetry codes as in Table 1 ▸), forming a tape structure along the *b*-axis direction. Adjacent tapes, which are related by a twofold rotation axis, are linked by a third C—H⋯O hydrogen bond (C13—H13⋯O6^ii^), forming wide ribbons parallel to the (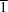
03) plane (Fig. 4 ▸). These ribbons are stacked *via* π–π inter­actions between the quinoline ring systems, forming layers parallel to the *ab* plane (Fig. 5 ▸). The centroid–centroid distances are 3.4935 (5), 3.6761 (6) and 3.7721 (6) Å, respectively, for *Cg*4⋯*Cg*4^iii^, *Cg*2⋯*Cg*2^iii^ and *Cg*2⋯*Cg*3^iii^, where *Cg*2, *Cg*3 and *Cg*4 are the centroids of the N2/C8–C11/C16, C11–C16 and N2/C8–C16 rings, respectively, of the base mol­ecule [symmetry code: (iii) −*x* + 1, *y*, −*z* + 2].

**Figure 6:**
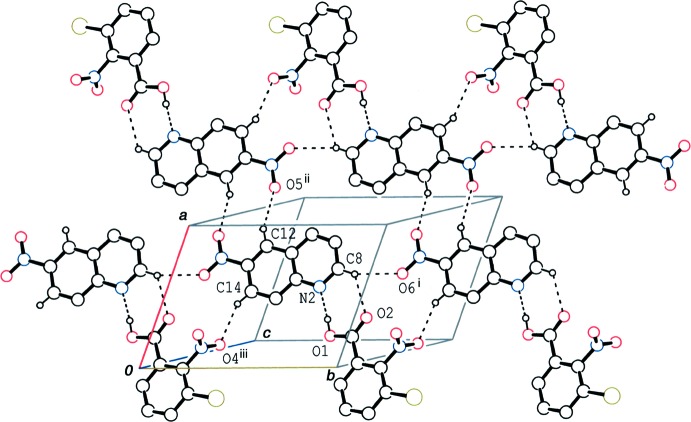
A packing diagram of (II), showing the ribbon structure along the *b*-axis direction formed by O—H⋯N and C—H⋯O hydrogen bonds (dashed lines). H atoms not involved in the hydrogen bonds have been omitted. [Symmetry codes: (i) *x*, *y* + 1, *z*; (ii) −*x* + 2, −*y*, −*z* + 1; (iii) *x*, *y* − 1, *z*.]

**Figure 7:**
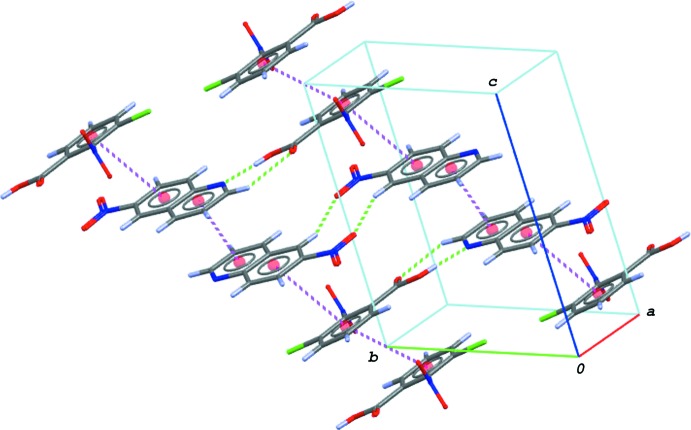
A partial packing diagram of (II), showing the column structure along [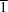
11] formed by weak π–π inter­actions (magenta dashed lines). The O—H⋯N and C—H⋯O hydrogen bonds in the hydrogen-bonded acid–base units are indicated by green dashed lines. The π–π inter­actions including the centroid of the ten-membered quinoline ring system (*Cg*4) are omitted for clarity.

In the crystal of (II), the hydrogen-bonded acid–base units are also linked into a tape structure along the *b*-axis direction *via* C—H⋯O hydrogen bonds (C8—H8⋯O6^i^ and C14—H14⋯O4^iii^; symmetry codes as in Table 2 ▸). Inversion-related tapes are linked by a further C—H⋯O hydrogen bond (C12—H12⋯O5^ii^; Table 2 ▸), forming wide ribbons parallel to the (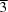
08) plane (Fig. 6 ▸). The acid and base mol­ecules are further stacked in a column along [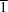
11] in an *⋯A⋯A⋯B⋯B⋯A⋯A⋯B⋯B⋯* manner (*A*: acid and *B*: base) *via* weak π–π inter­actions (Fig. 7 ▸), so forming a three-dimensional structure. The centroid–centroid distances are 3.8016 (8), 3.8666 (8), 3.9247 (9) and 3.8225 (8) Å, respectively, for *Cg*1⋯*Cg*1^iv^, *Cg*1⋯*Cg*3^v^, *Cg*2⋯*Cg*2^vi^ and *Cg*2⋯*Cg*4^vi^, where *Cg*1, *Cg*2, *Cg*3 and *Cg*4 are, respectively, the centroids of the C1–C6 ring of the acid mol­ecule, and the N2/C8–C11/C16, C11–C16 and N2/C8–C16 rings of the base mol­ecule [symmetry codes: (iv) −*x*, −*y* + 2, −*z*; (v) *x* − 1, *y* + 1, *z*; (vi) −*x* + 1, −*y* + 1, −*z* + 1]. A pair of short O⋯N contacts [O6⋯N3^vii^ = 2.8453 (13) Å; symmetry code: (vii) −*x* + 1, −*y*, −*x* + 1] between the nitro groups of the base mol­ecule are alsso observed.

**Figure 8:**
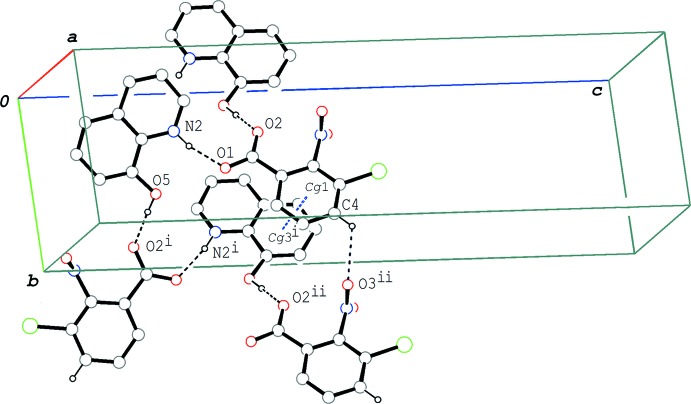
A partial packing diagram of (III), showing the 2_1_ helix running along the *b-*axis direction formed by O—H⋯O and N—H⋯O hydrogen bonds (black dashed lines). The C—H⋯O and π–π inter­actions observed in the chain are indicated by black and blue dashed lines, respectively. *Cg*1 and *Cg*3 are the centroids of the C1–C6 and C11–C16 rings, respectively. H atoms not involved in the hydrogen bonds have been omitted. [Symmetry codes: (i) −*x* + 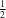, *y* + 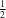, −*z* + 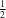; (ii) *x*, *y* + 1, *z*.]

**Figure 9:**
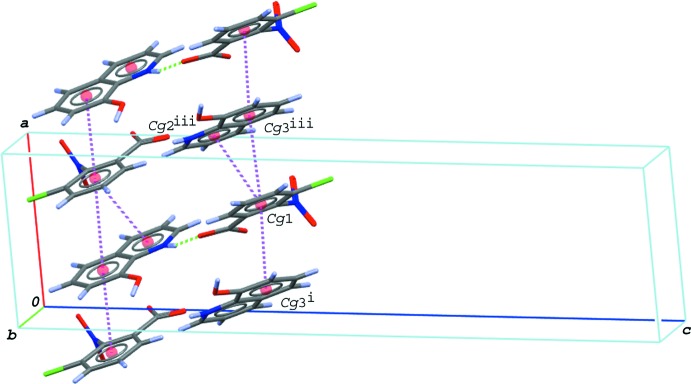
A partial packing diagram of (III), showing the π–π inter­actions (magenta dashed lines). The N—H⋯O hydrogen bonds in the hydrogen-bonded acid–base units are indicated by green dashed lines. *Cg*1, *Cg*2 and *Cg*3 are the centroids of the C1–C6, N2/C8–C11/C16 and C11–C16 rings, respectively. The π–π inter­actions including the centroid of the ten-membered quinoline ring system (*Cg*4) are omitted for clarity. [Symmetry codes: (i) −*x* + 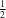, *y* + 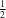, −*z* + 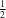; (ii) *x*, *y* + 1, *z*; (iii) −*x* + 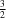, *y* + 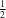, −*z* + 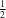.]

In the crystal of compound (III), the cations and the anions are alternately linked *via* N—H⋯O and O—H⋯O hydrogen bonds (N2—H2⋯O1 and O5—H5*O*⋯O2^i^; symmetry code as in Table 3 ▸), forming a 2_1_ helical chain running along the *b-*axis direction (Fig. 8 ▸). In the chain, a C—H⋯O (C4—H4⋯O3^ii^; Table 3 ▸) inter­action formed between the anions and a π–π inter­action between the C1–C6 ring and the C11–C16 ring are observed [*Cg*1⋯*Cg*3^i^ = 3.5570 (6) Å]; *Cg*1 and *Cg*3 are, respectively, the centroids of the C1–C6 ring of the anion and the C11–C16 ring of the cation. In addition to the π–π inter­action (*Cg*1⋯*Cg*3^i^), other π–π inter­actions are observed; the centroid–centroid distances are 3.5469 (6), 3.8550 (6) and 3.5133 (6) Å, respectively, for *Cg*1⋯*Cg*2^iii^, *Cg*1⋯*Cg*3^iii^ and *Cg*1⋯*Cg*4^iii^, where *Cg*2 and *Cg*4 are the centroids of the N2/C8–C11/C16 and N2/C8–C16 rings of the cation, respectively [symmetry code: (iii) −*x* + 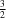, *y* + 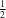, −*z* + 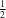]. The cations and the anions are stacked alternately in columns along the *a*-axis direction *via* the π–π inter­actions (Fig. 9 ▸), and the mol­ecular chains are linked into layers parallel to the *ab* plane through these inter­actions. A short Cl⋯O contact [Cl1⋯O3^iv^ = 3.0669 (10) Å; symmetry code: (iv) −*x* + 2, −*y* + 1, −*z* + 1] is observed between the layers.

## Database survey   

A search of the Cambridge Structural Database (Version 5.40, last update May 2019; Groom *et al.*, 2016 ▸) for organic co-crystals of 3-chloro-2-nitro­bonzoic acid with base mol­ecules gave six hits (five compounds), namely, 4-benzpyl­pyridine (1/1) (refcode UHAQUP; Sugiyama *et al.*, 2002 ▸), quinolone (1/1) (AJIWOG; Gotoh & Ishida, 2009 ▸), phthalazine (1/1) (CALJUW; Gotoh & Ishida, 2011 ▸), iso­quinoline (1/1) (NOVLAN; Gotoh & Ishida, 2015 ▸) and 4,4′-bi­pyridine (2/1) (XICGUO and XICGUO01; Rawat *et al.*, 2018 ▸). The structure of 3-chloro-2-nitro­bonzoic acid itself (XICHAV) was also reported by Rawat *et al.* (2018 ▸). There is no structure for a salt of 3-chloro-2-nitro­bonzoic acid with an organic base mol­ecule. In the acid mol­ecules of the above compounds, the dihedral angles between the benzene ring and the nitro group, and between the benzene ring and the carb­oxy group are in the ranges 79.1 (3)–89.9 (3)° and 1.4 (3)–14.2 (3)°, respectively, which agree with those in the three title compounds. The Δp*K*_a_ values for UHAQUP, AJIWOG, CALJUW, NOVLAN and XICGUO are 1.32, 3.08, 1.65, 3.58 and 3.27, respectively, and these compounds show short O⋯N distances in the O—H⋯N hydrogen bonds of 2.600 (3), 2.561 (1), 2.540 (2)–2.571 (2), 2.573 (1) and 2.613 (3) Å, respectively. Furthermore, in the short hydrogen bonds of AJIWOG, CALJUW and NOVLAN, the H atom is disordered over two positions. On the other hand, the compounds (I), (II) and (III) with Δp*K*_a_ values of 0.98, 1.42 and 3.02, respectively, show longer O⋯N distances of 2.673 (1), 2.631 (1) and 2.636 (1) Å, which suggests that the Δp*K*_a_ value is not an effective measure of hydrogen-bond strength in the 3-chloro-2-nitro­benzoic acid–organic base system.

A search for organic co-crystals/salts of 5-nitro­quinoline showed six structures. Limiting the search to benzoic acid derivatives gave two hits, namely, 3-amino­benzoic acid–5-nitro­quinoline (1/1) (PANYIM; Lynch *et al.*, 1997 ▸) and 4-animo­benzoic acid–5-nitro­quinoline (1/2) (PANZEJ; Lynch *et al.*, 1997 ▸). No structure was found in the CSD for organic co-crystals/salts of 6-nitro­quinoline. A search for organic co-crystals/salts of 8-hy­droxy­quinoline gave 17 hits. Of these compounds, one related compound is 8-hy­droxy­quinolinium 2-chloro-4-nitro­benzoate (WOPDEM; Babu & Chandrasekaran, 2014 ▸; Δp*K*_a_ = 2.80), in which the O⋯N distance of the N—H⋯O hydrogen bond is 2.644 (3) Å.

## Synthesis and crystallization   

Crystals of all three compounds, (I)–(III), were obtained by slow evaporation from aceto­nitrile solutions of 3-chloro-2-nitro­benzoic acid with quinoline derivatives in a 1:1 molar ratio at room temperature [100 ml aceto­nitrile solution of 3-chloro-2-nitro­benzoic acid (0.39 g) and 5-nitro­quinoline (0.34 g) for (I), 150 ml aceto­nitrile solution of 3-chloro-2-nitro­benzoic acid (0.45 g) and 6-nitro­quinoline (0.39 g) for (II), and 120 ml aceto­nitrile solution of 3-chloro-2-nitro­benzoic acid (0.55 g) and 8-hy­droxy­qunoline (0.40 mg) for (III)] .

## Refinement   

Crystal data, data collection and structure refinement details are summarized in Table 4 ▸. All H atoms in compounds (I)–(III) were found in difference-Fourier maps. O- and N-bound H atoms in (I)–(III) were refined freely [refined distances: O1—H1 = 0.88 (2) Å in (I), N1—H1 = 0.87 (3) Å in (II), and N2—H2 = 0.880 (16) and O5—H5*O* = 0.872 (19) Å in (III),]. Other H atoms were positioned geometrically (C—H = 0.95 Å) and treated as riding, with *U*_iso_(H) = 1.2*U*_eq_(C).

## Supplementary Material

Crystal structure: contains datablock(s) global, I, II, III. DOI: 10.1107/S2056989019012799/lh5922sup1.cif

Structure factors: contains datablock(s) I. DOI: 10.1107/S2056989019012799/lh5922Isup2.hkl

Structure factors: contains datablock(s) II. DOI: 10.1107/S2056989019012799/lh5922IIsup3.hkl

Structure factors: contains datablock(s) III. DOI: 10.1107/S2056989019012799/lh5922IIIsup4.hkl

CCDC references: 1953605, 1953604, 1953603

Additional supporting information:  crystallographic information; 3D view; checkCIF report

## Figures and Tables

**Table 1:** Hydrogen-bond geometry (Å, °) for (I)

*D*—H⋯*A*	*D*—H	H⋯*A*	*D*⋯*A*	*D*—H⋯*A*
O1—H1⋯N2	0.88 (2)	1.80 (2)	2.6727 (12)	178 (2)
C8—H8⋯O2	0.95	2.48	3.1820 (13)	131
C5—H5⋯O2^i^	0.95	2.57	3.4860 (14)	163
C14—H14⋯O5^i^	0.95	2.56	3.4644 (14)	159
C13—H13⋯O6^ii^	0.95	2.32	3.1495 (14)	146

Symmetry codes: (i) 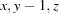; (ii) 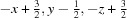.

**Table 2:** Hydrogen-bond geometry (Å, °) for (II)

*D*—H⋯*A*	*D*—H	H⋯*A*	*D*⋯*A*	*D*—H⋯*A*
O1—H1⋯N2	0.87 (3)	1.76 (3)	2.6310 (14)	176 (3)
C8—H8⋯O2	0.95	2.53	3.2082 (16)	128
C8—H8⋯O6^i^	0.95	2.41	3.2387 (15)	145
C12—H12⋯O5^ii^	0.95	2.37	3.2526 (16)	155
C14—H14⋯O4^iii^	0.95	2.52	3.3226 (16)	142

Symmetry codes: (i) 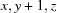; (ii) 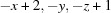; (iii) 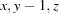.

**Table 3:** Hydrogen-bond geometry (Å, °) for (III)

*D*—H⋯*A*	*D*—H	H⋯*A*	*D*⋯*A*	*D*—H⋯*A*
N2—H2⋯O1	0.880 (16)	1.776 (16)	2.6355 (12)	164.7 (14)
O5—H5*O*⋯O2^i^	0.872 (19)	1.756 (19)	2.6247 (12)	173.2 (19)
C4—H4⋯O3^ii^	0.95	2.49	3.1082 (12)	123

Symmetry codes: (i) 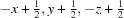; (ii) 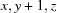.

**Table 4:** Experimental details

	(I)	(II)	(III)
Crystal data			
Chemical formula	C_7_H_4_ClNO_4_·C_9_H_6_N_2_O_2_	C_7_H_4_ClNO_4_·C_9_H_6_N_2_O_2_	C_7_H_3_ClNO_4_·C_9_H_8_NO
*M*_r_	375.72	375.72	346.73
Crystal system, space group	Monoclinic, *C*2/*c*	Triclinic, *P* 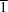	Monoclinic, *P*2_1_/*n*
Temperature (K)	190	190	190
*a*, *b*, *c* (Å)	20.5876 (4), 7.6889 (3), 20.4312 (4)	7.7282 (10), 10.2839 (14), 11.2828 (16)	7.3409 (5), 7.4689 (4), 27.0427 (14)
α, β, γ (°)	90, 104.5338 (7), 90	71.990 (4), 79.724 (4), 69.051 (3)	90, 95.7158 (19), 90
*V* (Å^3^)	3130.70 (16)	794.08 (19)	1475.33 (15)
*Z*	8	2	4
Radiation type	Mo *K*α	Mo *K*α	Mo *K*α
μ (mm^−1^)	0.29	0.28	0.29
Crystal size (mm)	0.45 × 0.40 × 0.30	0.38 × 0.35 × 0.30	0.45 × 0.30 × 0.26

Data collection			
Diffractometer	Rigaku R-AXIS RAPIDII	Rigaku R-AXIS RAPIDII	Rigaku R-AXIS RAPIDII
Absorption correction	Numerical (*NUMABS*; Higashi, 1999 ▸)	Numerical (*NUMABS*; Higashi, 1999 ▸)	Numerical (*NUMABS*; Higashi, 1999 ▸)
*T*_min_, *T*_max_	0.837, 0.918	0.887, 0.919	0.844, 0.927
No. of measured, independent and observed [*I* > 2σ(*I*)] reflections	30107, 4549, 4077	16549, 4622, 4029	29560, 4311, 3937
*R*_int_	0.022	0.026	0.019
(sin θ/λ)_max_ (Å^−1^)	0.703	0.703	0.703

Refinement			
*R*[*F* ^2^ > 2σ(*F* ^2^)], *wR*(*F* ^2^), *S*	0.037, 0.104, 1.05	0.039, 0.113, 1.07	0.033, 0.092, 1.06
No. of reflections	4549	4622	4311
No. of parameters	239	239	225
H-atom treatment	H atoms treated by a mixture of independent and constrained refinement	H atoms treated by a mixture of independent and constrained refinement	H atoms treated by a mixture of independent and constrained refinement
Δρ_max_, Δρ_min_ (e Å^−3^)	0.30, −0.35	0.50, −0.33	0.39, −0.30

Computer programs: *RAPID-AUTO* (Rigaku, 2006 ▸), *SHELXT2018/2* (Sheldrick, 2015*a* ▸), *SHELXL2018/3* (Sheldrick, 2015*b* ▸), *ORTEP-3 for Windows* (Farrugia, 2012 ▸) and *Mercury* (Macrae *et al.*, 2006 ▸), *CrystalStructure* (Rigaku, 2018 ▸) and *PLATON* (Spek, 2015 ▸).

## supplementary crystallographic information

### 3-Chloro-2-nitrobenzoic acid–5-nitroquinoline (1/1) (I) Crystal data

**Table:**

C_7_H_4_ClNO_4_·C_9_H_6_N_2_O_2_	*F*(000) = 1536.00
*M**_r_* = 375.72	*D*_x_ = 1.594 Mg m^−^^3^
Monoclinic, *C*2/*c*	Mo *K*α radiation, λ = 0.71075 Å
*a* = 20.5876 (4) Å	Cell parameters from 25710 reflections
*b* = 7.6889 (3) Å	θ = 3.1–30.1°
*c* = 20.4312 (4) Å	µ = 0.29 mm^−^^1^
β = 104.5338 (7)°	*T* = 190 K
*V* = 3130.70 (16) Å^3^	Block, colorless
*Z* = 8	0.45 × 0.40 × 0.30 mm

### 3-Chloro-2-nitrobenzoic acid–5-nitroquinoline (1/1) (I) Data collection

**Table:**

Rigaku R-AXIS RAPIDII diffractometer	4077 reflections with *I* > 2σ(*I*)
Detector resolution: 10.000 pixels mm^-1^	*R*_int_ = 0.022
ω scans	θ_max_ = 30.0°, θ_min_ = 3.1°
Absorption correction: numerical (*NUMABS*; Higashi, 1999)	*h* = −26→28
*T*_min_ = 0.837, *T*_max_ = 0.918	*k* = −10→10
30107 measured reflections	*l* = −28→28
4549 independent reflections	

### 3-Chloro-2-nitrobenzoic acid–5-nitroquinoline (1/1) (I) Refinement

**Table:**

Refinement on *F*^2^	Primary atom site location: structure-invariant direct methods
Least-squares matrix: full	Secondary atom site location: difference Fourier map
*R*[*F*^2^ > 2σ(*F*^2^)] = 0.037	Hydrogen site location: mixed
*wR*(*F*^2^) = 0.104	H atoms treated by a mixture of independent and constrained refinement
*S* = 1.05	*w* = 1/[σ^2^(*F*_o_^2^) + (0.0636*P*)^2^ + 1.2587*P*] where *P* = (*F*_o_^2^ + 2*F*_c_^2^)/3
4549 reflections	(Δ/σ)_max_ = 0.001
239 parameters	Δρ_max_ = 0.30 e Å^−^^3^
0 restraints	Δρ_min_ = −0.35 e Å^−^^3^

### 3-Chloro-2-nitrobenzoic acid–5-nitroquinoline (1/1) (I) Special details

**Table:**

Geometry. All esds (except the esd in the dihedral angle between two l.s. planes) are estimated using the full covariance matrix. The cell esds are taken into account individually in the estimation of esds in distances, angles and torsion angles; correlations between esds in cell parameters are only used when they are defined by crystal symmetry. An approximate (isotropic) treatment of cell esds is used for estimating esds involving l.s. planes.

### 3-Chloro-2-nitrobenzoic acid–5-nitroquinoline (1/1) (I) Fractional atomic coordinates and isotropic or equivalent isotropic displacement parameters (Å^2^)

**Table:**

	*x*	*y*	*z*	*U*_iso_*/*U*_eq_	
Cl1	0.03434 (2)	0.24994 (4)	0.50043 (2)	0.03753 (9)	
O1	0.34832 (4)	0.34621 (10)	0.61778 (4)	0.03292 (17)	
O2	0.27238 (4)	0.55880 (10)	0.60848 (5)	0.03766 (19)	
O3	0.14558 (5)	0.57618 (11)	0.50150 (4)	0.0412 (2)	
O4	0.12329 (5)	0.55420 (11)	0.59855 (5)	0.0414 (2)	
O5	0.64633 (4)	0.97971 (11)	0.75715 (4)	0.03764 (19)	
O6	0.72372 (4)	0.82403 (14)	0.73078 (5)	0.0472 (2)	
N1	0.14396 (4)	0.49673 (11)	0.55218 (5)	0.02666 (17)	
N2	0.43699 (4)	0.60692 (11)	0.64264 (4)	0.02613 (17)	
N3	0.66566 (4)	0.84775 (12)	0.73417 (4)	0.02984 (18)	
C1	0.23369 (5)	0.26976 (12)	0.58259 (5)	0.02307 (18)	
C2	0.16614 (4)	0.31430 (12)	0.55708 (5)	0.02215 (17)	
C3	0.11699 (5)	0.18860 (13)	0.53478 (5)	0.02620 (19)	
C4	0.13470 (5)	0.01433 (14)	0.53907 (6)	0.0320 (2)	
H4	0.101547	−0.072633	0.523966	0.038*	
C5	0.20130 (6)	−0.03225 (14)	0.56561 (6)	0.0344 (2)	
H5	0.213441	−0.151718	0.569282	0.041*	
C6	0.25043 (5)	0.09415 (13)	0.58689 (6)	0.0296 (2)	
H6	0.295828	0.060186	0.604524	0.035*	
C7	0.28632 (5)	0.40747 (12)	0.60433 (5)	0.02479 (18)	
C8	0.41075 (5)	0.76300 (13)	0.63187 (5)	0.0281 (2)	
H8	0.363528	0.772862	0.614770	0.034*	
C9	0.44900 (5)	0.91633 (13)	0.64438 (5)	0.0290 (2)	
H9	0.427911	1.026448	0.634382	0.035*	
C10	0.51692 (5)	0.90588 (12)	0.67109 (5)	0.02615 (19)	
H10	0.543243	1.008630	0.680454	0.031*	
C11	0.54753 (5)	0.74008 (11)	0.68461 (5)	0.02182 (17)	
C12	0.61745 (5)	0.70585 (13)	0.71097 (5)	0.02475 (18)	
C13	0.64371 (5)	0.54231 (14)	0.71854 (6)	0.0311 (2)	
H13	0.690681	0.525741	0.735325	0.037*	
C14	0.60078 (6)	0.39830 (14)	0.70133 (6)	0.0356 (2)	
H14	0.618748	0.283873	0.706670	0.043*	
C15	0.53282 (6)	0.42258 (13)	0.67677 (6)	0.0313 (2)	
H15	0.504064	0.324562	0.665699	0.038*	
C16	0.50536 (5)	0.59203 (12)	0.66783 (5)	0.02310 (18)	
H1	0.3780 (11)	0.430 (3)	0.6264 (10)	0.073 (6)*	

### 3-Chloro-2-nitrobenzoic acid–5-nitroquinoline (1/1) (I) Atomic displacement parameters (Å^2^)

**Table:**

	*U*^11^	*U*^22^	*U*^33^	*U*^12^	*U*^13^	*U*^23^
Cl1	0.02113 (13)	0.03817 (16)	0.04799 (17)	−0.00501 (9)	−0.00126 (11)	−0.00276 (10)
O1	0.0197 (3)	0.0265 (4)	0.0495 (5)	−0.0024 (3)	0.0029 (3)	−0.0014 (3)
O2	0.0245 (4)	0.0230 (3)	0.0615 (5)	−0.0035 (3)	0.0033 (3)	−0.0040 (3)
O3	0.0537 (5)	0.0289 (4)	0.0359 (4)	0.0016 (3)	0.0018 (4)	0.0084 (3)
O4	0.0421 (5)	0.0322 (4)	0.0559 (5)	0.0007 (3)	0.0237 (4)	−0.0094 (4)
O5	0.0346 (4)	0.0320 (4)	0.0431 (4)	−0.0071 (3)	0.0037 (3)	−0.0078 (3)
O6	0.0214 (4)	0.0566 (6)	0.0631 (6)	−0.0097 (4)	0.0097 (4)	−0.0088 (5)
N1	0.0211 (4)	0.0221 (4)	0.0344 (4)	−0.0018 (3)	0.0025 (3)	−0.0014 (3)
N2	0.0195 (4)	0.0268 (4)	0.0299 (4)	−0.0039 (3)	0.0020 (3)	0.0007 (3)
N3	0.0221 (4)	0.0337 (4)	0.0314 (4)	−0.0067 (3)	0.0022 (3)	−0.0007 (3)
C1	0.0209 (4)	0.0210 (4)	0.0265 (4)	−0.0031 (3)	0.0044 (3)	−0.0008 (3)
C2	0.0215 (4)	0.0196 (4)	0.0248 (4)	−0.0018 (3)	0.0047 (3)	−0.0002 (3)
C3	0.0216 (4)	0.0260 (4)	0.0294 (4)	−0.0046 (3)	0.0036 (3)	−0.0019 (3)
C4	0.0300 (5)	0.0237 (5)	0.0405 (5)	−0.0075 (4)	0.0057 (4)	−0.0040 (4)
C5	0.0341 (5)	0.0196 (4)	0.0477 (6)	−0.0026 (4)	0.0068 (5)	−0.0018 (4)
C6	0.0259 (4)	0.0221 (4)	0.0391 (5)	0.0000 (3)	0.0050 (4)	−0.0001 (4)
C7	0.0200 (4)	0.0239 (4)	0.0290 (4)	−0.0032 (3)	0.0035 (3)	0.0002 (3)
C8	0.0197 (4)	0.0304 (5)	0.0321 (5)	−0.0014 (3)	0.0025 (4)	0.0042 (4)
C9	0.0245 (4)	0.0241 (4)	0.0369 (5)	0.0014 (3)	0.0053 (4)	0.0051 (4)
C10	0.0230 (4)	0.0216 (4)	0.0332 (5)	−0.0019 (3)	0.0059 (4)	0.0017 (3)
C11	0.0186 (4)	0.0219 (4)	0.0243 (4)	−0.0020 (3)	0.0042 (3)	−0.0004 (3)
C12	0.0187 (4)	0.0275 (4)	0.0267 (4)	−0.0030 (3)	0.0032 (3)	−0.0015 (3)
C13	0.0223 (4)	0.0319 (5)	0.0368 (5)	0.0049 (4)	0.0032 (4)	0.0004 (4)
C14	0.0325 (5)	0.0246 (5)	0.0470 (6)	0.0061 (4)	0.0047 (5)	−0.0010 (4)
C15	0.0287 (5)	0.0218 (4)	0.0409 (5)	−0.0016 (3)	0.0038 (4)	−0.0022 (4)
C16	0.0203 (4)	0.0220 (4)	0.0259 (4)	−0.0027 (3)	0.0037 (3)	−0.0007 (3)

### 3-Chloro-2-nitrobenzoic acid–5-nitroquinoline (1/1) (I) Geometric parameters (Å, º)

**Table:**

Cl1—C3	1.7352 (10)	C5—C6	1.3913 (14)
O1—C7	1.3231 (12)	C5—H5	0.9500
O1—H1	0.88 (2)	C6—H6	0.9500
O2—C7	1.2065 (13)	C8—C9	1.4051 (14)
O3—N1	1.2099 (12)	C8—H8	0.9500
O4—N1	1.2150 (12)	C9—C10	1.3699 (13)
O5—N3	1.2256 (13)	C9—H9	0.9500
O6—N3	1.2282 (12)	C10—C11	1.4182 (13)
N1—C2	1.4708 (12)	C10—H10	0.9500
N2—C8	1.3118 (13)	C11—C16	1.4205 (12)
N2—C16	1.3768 (12)	C11—C12	1.4290 (13)
N3—C12	1.4711 (13)	C12—C13	1.3621 (14)
C1—C6	1.3909 (13)	C13—C14	1.4053 (16)
C1—C2	1.3997 (13)	C13—H13	0.9500
C1—C7	1.5002 (13)	C14—C15	1.3755 (16)
C2—C3	1.3909 (12)	C14—H14	0.9500
C3—C4	1.3857 (14)	C15—C16	1.4138 (13)
C4—C5	1.3889 (16)	C15—H15	0.9500
C4—H4	0.9500		
			
C7—O1—H1	111.7 (14)	O1—C7—C1	113.43 (8)
O3—N1—O4	125.03 (10)	N2—C8—C9	123.28 (9)
O3—N1—C2	117.71 (9)	N2—C8—H8	118.4
O4—N1—C2	117.21 (9)	C9—C8—H8	118.4
C8—N2—C16	118.56 (8)	C10—C9—C8	119.51 (9)
O5—N3—O6	123.97 (9)	C10—C9—H9	120.2
O5—N3—C12	118.67 (8)	C8—C9—H9	120.2
O6—N3—C12	117.33 (9)	C9—C10—C11	119.32 (9)
C6—C1—C2	117.92 (8)	C9—C10—H10	120.3
C6—C1—C7	121.16 (9)	C11—C10—H10	120.3
C2—C1—C7	120.92 (8)	C10—C11—C16	117.28 (8)
C3—C2—C1	121.68 (9)	C10—C11—C12	126.54 (8)
C3—C2—N1	116.90 (8)	C16—C11—C12	116.12 (8)
C1—C2—N1	121.41 (8)	C13—C12—C11	123.12 (9)
C4—C3—C2	119.53 (9)	C13—C12—N3	115.57 (9)
C4—C3—Cl1	120.28 (7)	C11—C12—N3	121.28 (9)
C2—C3—Cl1	120.18 (8)	C12—C13—C14	119.48 (9)
C3—C4—C5	119.50 (9)	C12—C13—H13	120.3
C3—C4—H4	120.3	C14—C13—H13	120.3
C5—C4—H4	120.3	C15—C14—C13	120.17 (10)
C4—C5—C6	120.72 (10)	C15—C14—H14	119.9
C4—C5—H5	119.6	C13—C14—H14	119.9
C6—C5—H5	119.6	C14—C15—C16	120.63 (9)
C1—C6—C5	120.61 (10)	C14—C15—H15	119.7
C1—C6—H6	119.7	C16—C15—H15	119.7
C5—C6—H6	119.7	N2—C16—C15	117.59 (8)
O2—C7—O1	124.25 (9)	N2—C16—C11	121.96 (8)
O2—C7—C1	122.32 (9)	C15—C16—C11	120.46 (9)
			
C6—C1—C2—C3	−1.77 (14)	C8—C9—C10—C11	1.01 (15)
C7—C1—C2—C3	178.02 (9)	C9—C10—C11—C16	1.49 (14)
C6—C1—C2—N1	178.69 (9)	C9—C10—C11—C12	178.64 (9)
C7—C1—C2—N1	−1.53 (14)	C10—C11—C12—C13	−175.60 (10)
O3—N1—C2—C3	−92.47 (11)	C16—C11—C12—C13	1.57 (14)
O4—N1—C2—C3	85.14 (11)	C10—C11—C12—N3	6.67 (15)
O3—N1—C2—C1	87.09 (12)	C16—C11—C12—N3	−176.16 (8)
O4—N1—C2—C1	−95.29 (11)	O5—N3—C12—C13	−148.03 (10)
C1—C2—C3—C4	1.39 (15)	O6—N3—C12—C13	30.14 (14)
N1—C2—C3—C4	−179.05 (9)	O5—N3—C12—C11	29.86 (14)
C1—C2—C3—Cl1	−177.45 (7)	O6—N3—C12—C11	−151.97 (10)
N1—C2—C3—Cl1	2.11 (12)	C11—C12—C13—C14	−1.45 (16)
C2—C3—C4—C5	0.03 (16)	N3—C12—C13—C14	176.40 (10)
Cl1—C3—C4—C5	178.87 (9)	C12—C13—C14—C15	0.27 (18)
C3—C4—C5—C6	−1.02 (18)	C13—C14—C15—C16	0.68 (18)
C2—C1—C6—C5	0.76 (16)	C8—N2—C16—C15	−177.79 (10)
C7—C1—C6—C5	−179.03 (10)	C8—N2—C16—C11	2.30 (14)
C4—C5—C6—C1	0.62 (18)	C14—C15—C16—N2	179.60 (10)
C6—C1—C7—O2	−171.24 (11)	C14—C15—C16—C11	−0.50 (16)
C2—C1—C7—O2	8.99 (15)	C10—C11—C16—N2	−3.23 (14)
C6—C1—C7—O1	9.12 (14)	C12—C11—C16—N2	179.32 (8)
C2—C1—C7—O1	−170.66 (9)	C10—C11—C16—C15	176.87 (9)
C16—N2—C8—C9	0.41 (16)	C12—C11—C16—C15	−0.58 (14)
N2—C8—C9—C10	−2.09 (17)		

### 3-Chloro-2-nitrobenzoic acid–5-nitroquinoline (1/1) (I) Hydrogen-bond geometry (Å, º)

**Table:**

*D*—H···*A*	*D*—H	H···*A*	*D*···*A*	*D*—H···*A*
O1—H1···N2	0.88 (2)	1.80 (2)	2.6727 (12)	178 (2)
C8—H8···O2	0.95	2.48	3.1820 (13)	131
C5—H5···O2^i^	0.95	2.57	3.4860 (14)	163
C14—H14···O5^i^	0.95	2.56	3.4644 (14)	159
C13—H13···O6^ii^	0.95	2.32	3.1495 (14)	146

Symmetry codes: (i) *x*, *y*−1, *z*; (ii) −*x*+3/2, *y*−1/2, −*z*+3/2.

### 3-Chloro-2-nitrobenzoicacid–6-nitroquinoline (1/1) (II) Crystal data

**Table:**

C_7_H_4_ClNO_4_·C_9_H_6_N_2_O_2_	*Z* = 2
*M**_r_* = 375.72	*F*(000) = 384.00
Triclinic, *P*1	*D*_x_ = 1.571 Mg m^−^^3^
*a* = 7.7282 (10) Å	Mo *K*α radiation, λ = 0.71075 Å
*b* = 10.2839 (14) Å	Cell parameters from 14524 reflections
*c* = 11.2828 (16) Å	θ = 3.1–30.1°
α = 71.990 (4)°	µ = 0.28 mm^−^^1^
β = 79.724 (4)°	*T* = 190 K
γ = 69.051 (3)°	Block, colorless
*V* = 794.08 (19) Å^3^	0.38 × 0.35 × 0.30 mm

### 3-Chloro-2-nitrobenzoicacid–6-nitroquinoline (1/1) (II) Data collection

**Table:**

Rigaku R-AXIS RAPIDII diffractometer	4029 reflections with *I* > 2σ(*I*)
Detector resolution: 10.000 pixels mm^-1^	*R*_int_ = 0.026
ω scans	θ_max_ = 30.0°, θ_min_ = 3.1°
Absorption correction: numerical (*NUMABS*; Higashi, 1999)	*h* = −10→10
*T*_min_ = 0.887, *T*_max_ = 0.919	*k* = −14→14
16549 measured reflections	*l* = −15→15
4622 independent reflections	

### 3-Chloro-2-nitrobenzoicacid–6-nitroquinoline (1/1) (II) Refinement

**Table:**

Refinement on *F*^2^	Primary atom site location: structure-invariant direct methods
Least-squares matrix: full	Secondary atom site location: difference Fourier map
*R*[*F*^2^ > 2σ(*F*^2^)] = 0.039	Hydrogen site location: mixed
*wR*(*F*^2^) = 0.113	H atoms treated by a mixture of independent and constrained refinement
*S* = 1.07	*w* = 1/[σ^2^(*F*_o_^2^) + (0.072*P*)^2^ + 0.0976*P*] where *P* = (*F*_o_^2^ + 2*F*_c_^2^)/3
4622 reflections	(Δ/σ)_max_ = 0.001
239 parameters	Δρ_max_ = 0.50 e Å^−^^3^
0 restraints	Δρ_min_ = −0.33 e Å^−^^3^

### 3-Chloro-2-nitrobenzoicacid–6-nitroquinoline (1/1) (II) Special details

**Table:**

Geometry. All esds (except the esd in the dihedral angle between two l.s. planes) are estimated using the full covariance matrix. The cell esds are taken into account individually in the estimation of esds in distances, angles and torsion angles; correlations between esds in cell parameters are only used when they are defined by crystal symmetry. An approximate (isotropic) treatment of cell esds is used for estimating esds involving l.s. planes.

### 3-Chloro-2-nitrobenzoicacid–6-nitroquinoline (1/1) (II) Fractional atomic coordinates and isotropic or equivalent isotropic displacement parameters (Å^2^)

**Table:**

	*x*	*y*	*z*	*U*_iso_*/*U*_eq_	
Cl1	−0.21217 (4)	1.44247 (3)	−0.00166 (3)	0.04778 (11)	
O1	0.17321 (12)	0.77718 (8)	0.24868 (9)	0.03883 (19)	
O2	0.30609 (12)	0.94833 (9)	0.20949 (10)	0.0464 (2)	
O3	0.23107 (11)	1.23134 (10)	0.01395 (9)	0.0439 (2)	
O4	0.12561 (13)	1.25965 (10)	0.19770 (10)	0.0477 (2)	
O5	0.83957 (13)	−0.07571 (9)	0.45563 (10)	0.0469 (2)	
O6	0.58065 (12)	−0.04120 (8)	0.38490 (8)	0.03651 (18)	
N1	0.12435 (12)	1.22061 (9)	0.10689 (9)	0.03234 (19)	
N2	0.50541 (13)	0.60187 (9)	0.31357 (9)	0.03239 (19)	
N3	0.69185 (13)	0.00332 (9)	0.41169 (8)	0.03128 (18)	
C1	−0.00331 (13)	1.01631 (10)	0.15345 (9)	0.02609 (18)	
C2	−0.02570 (12)	1.16317 (10)	0.10624 (9)	0.02667 (18)	
C3	−0.18851 (13)	1.26120 (10)	0.05510 (10)	0.0299 (2)	
C4	−0.33392 (14)	1.21363 (12)	0.04976 (10)	0.0325 (2)	
H4	−0.445873	1.280397	0.015146	0.039*	
C5	−0.31344 (14)	1.06810 (12)	0.09541 (10)	0.0318 (2)	
H5	−0.411793	1.034696	0.091986	0.038*	
C6	−0.14952 (13)	0.97031 (11)	0.14634 (9)	0.02906 (19)	
H6	−0.137031	0.870569	0.176756	0.035*	
C7	0.17485 (14)	0.91108 (10)	0.20737 (10)	0.0302 (2)	
C8	0.62786 (17)	0.65774 (11)	0.32741 (12)	0.0382 (2)	
H8	0.593373	0.759863	0.309365	0.046*	
C9	0.80676 (17)	0.57424 (13)	0.36745 (13)	0.0413 (3)	
H9	0.890518	0.619663	0.374699	0.050*	
C10	0.85796 (16)	0.42727 (12)	0.39567 (12)	0.0367 (2)	
H10	0.977047	0.368770	0.424193	0.044*	
C11	0.73037 (14)	0.36326 (10)	0.38169 (9)	0.02805 (19)	
C12	0.77423 (14)	0.21193 (10)	0.40730 (10)	0.02926 (19)	
H12	0.890452	0.148054	0.437393	0.035*	
C13	0.64508 (14)	0.16024 (10)	0.38761 (9)	0.02718 (19)	
C14	0.47039 (14)	0.24888 (11)	0.34310 (10)	0.0302 (2)	
H14	0.385636	0.207883	0.329622	0.036*	
C15	0.42557 (14)	0.39522 (11)	0.31976 (10)	0.0310 (2)	
H15	0.307926	0.457014	0.290674	0.037*	
C16	0.55426 (13)	0.45484 (10)	0.33887 (9)	0.02686 (18)	
H1	0.285 (3)	0.723 (3)	0.270 (2)	0.089 (7)*	

### 3-Chloro-2-nitrobenzoicacid–6-nitroquinoline (1/1) (II) Atomic displacement parameters (Å^2^)

**Table:**

	*U*^11^	*U*^22^	*U*^33^	*U*^12^	*U*^13^	*U*^23^
Cl1	0.03623 (16)	0.02349 (14)	0.0773 (2)	−0.00733 (10)	−0.02054 (14)	0.00154 (12)
O1	0.0321 (4)	0.0222 (3)	0.0574 (5)	−0.0076 (3)	−0.0081 (3)	−0.0028 (3)
O2	0.0339 (4)	0.0274 (4)	0.0765 (6)	−0.0091 (3)	−0.0234 (4)	−0.0020 (4)
O3	0.0315 (4)	0.0410 (4)	0.0603 (5)	−0.0196 (3)	−0.0013 (4)	−0.0068 (4)
O4	0.0429 (5)	0.0451 (5)	0.0664 (6)	−0.0136 (4)	−0.0170 (4)	−0.0240 (4)
O5	0.0430 (4)	0.0235 (4)	0.0679 (6)	−0.0034 (3)	−0.0177 (4)	−0.0047 (4)
O6	0.0471 (4)	0.0304 (4)	0.0395 (4)	−0.0196 (3)	−0.0002 (3)	−0.0132 (3)
N1	0.0246 (4)	0.0221 (4)	0.0508 (5)	−0.0070 (3)	−0.0109 (4)	−0.0065 (3)
N2	0.0329 (4)	0.0204 (4)	0.0417 (5)	−0.0051 (3)	−0.0079 (3)	−0.0066 (3)
N3	0.0368 (4)	0.0225 (4)	0.0340 (4)	−0.0100 (3)	−0.0023 (3)	−0.0066 (3)
C1	0.0231 (4)	0.0236 (4)	0.0311 (4)	−0.0078 (3)	−0.0029 (3)	−0.0060 (3)
C2	0.0217 (4)	0.0244 (4)	0.0349 (4)	−0.0085 (3)	−0.0041 (3)	−0.0071 (3)
C3	0.0250 (4)	0.0238 (4)	0.0389 (5)	−0.0071 (3)	−0.0065 (4)	−0.0042 (4)
C4	0.0220 (4)	0.0342 (5)	0.0401 (5)	−0.0080 (4)	−0.0063 (4)	−0.0075 (4)
C5	0.0255 (4)	0.0365 (5)	0.0374 (5)	−0.0152 (4)	−0.0011 (4)	−0.0099 (4)
C6	0.0273 (4)	0.0282 (4)	0.0335 (4)	−0.0132 (4)	0.0008 (3)	−0.0075 (4)
C7	0.0287 (4)	0.0229 (4)	0.0370 (5)	−0.0068 (3)	−0.0050 (4)	−0.0054 (3)
C8	0.0433 (6)	0.0228 (4)	0.0506 (6)	−0.0102 (4)	−0.0097 (5)	−0.0097 (4)
C9	0.0420 (6)	0.0310 (5)	0.0588 (7)	−0.0157 (4)	−0.0144 (5)	−0.0122 (5)
C10	0.0328 (5)	0.0294 (5)	0.0518 (6)	−0.0100 (4)	−0.0144 (4)	−0.0099 (4)
C11	0.0282 (4)	0.0229 (4)	0.0343 (5)	−0.0078 (3)	−0.0074 (3)	−0.0071 (3)
C12	0.0269 (4)	0.0218 (4)	0.0373 (5)	−0.0048 (3)	−0.0091 (4)	−0.0054 (3)
C13	0.0303 (4)	0.0204 (4)	0.0307 (4)	−0.0082 (3)	−0.0044 (3)	−0.0055 (3)
C14	0.0298 (4)	0.0270 (4)	0.0359 (5)	−0.0115 (4)	−0.0083 (4)	−0.0055 (4)
C15	0.0270 (4)	0.0258 (4)	0.0385 (5)	−0.0068 (3)	−0.0103 (4)	−0.0041 (4)
C16	0.0275 (4)	0.0210 (4)	0.0309 (4)	−0.0064 (3)	−0.0054 (3)	−0.0053 (3)

### 3-Chloro-2-nitrobenzoicacid–6-nitroquinoline (1/1) (II) Geometric parameters (Å, º)

**Table:**

Cl1—C3	1.7257 (10)	C5—C6	1.3921 (14)
O1—C7	1.3142 (12)	C5—H5	0.9500
O1—H1	0.87 (3)	C6—H6	0.9500
O2—C7	1.2117 (13)	C8—C9	1.4096 (16)
O3—N1	1.2153 (13)	C8—H8	0.9500
O4—N1	1.2132 (13)	C9—C10	1.3646 (15)
O5—N3	1.2243 (12)	C9—H9	0.9500
O6—N3	1.2244 (12)	C10—C11	1.4193 (14)
N1—C2	1.4796 (12)	C10—H10	0.9500
N2—C8	1.3218 (14)	C11—C12	1.4142 (13)
N2—C16	1.3688 (12)	C11—C16	1.4175 (13)
N3—C13	1.4682 (12)	C12—C13	1.3637 (14)
C1—C2	1.3940 (13)	C12—H12	0.9500
C1—C6	1.3947 (13)	C13—C14	1.4088 (13)
C1—C7	1.5044 (13)	C14—C15	1.3671 (14)
C2—C3	1.3873 (13)	C14—H14	0.9500
C3—C4	1.3934 (13)	C15—C16	1.4163 (14)
C4—C5	1.3832 (15)	C15—H15	0.9500
C4—H4	0.9500		
			
C7—O1—H1	107.0 (16)	O1—C7—C1	113.28 (9)
O4—N1—O3	125.19 (9)	N2—C8—C9	123.66 (10)
O4—N1—C2	117.34 (9)	N2—C8—H8	118.2
O3—N1—C2	117.37 (9)	C9—C8—H8	118.2
C8—N2—C16	118.54 (9)	C10—C9—C8	119.08 (10)
O5—N3—O6	123.52 (9)	C10—C9—H9	120.5
O5—N3—C13	118.83 (9)	C8—C9—H9	120.5
O6—N3—C13	117.64 (9)	C9—C10—C11	118.90 (10)
C2—C1—C6	117.80 (9)	C9—C10—H10	120.5
C2—C1—C7	120.79 (8)	C11—C10—H10	120.5
C6—C1—C7	121.39 (8)	C12—C11—C16	119.20 (9)
C3—C2—C1	121.41 (9)	C12—C11—C10	122.22 (9)
C3—C2—N1	117.24 (8)	C16—C11—C10	118.57 (9)
C1—C2—N1	121.35 (8)	C13—C12—C11	118.16 (9)
C2—C3—C4	120.06 (9)	C13—C12—H12	120.9
C2—C3—Cl1	120.21 (7)	C11—C12—H12	120.9
C4—C3—Cl1	119.73 (8)	C12—C13—C14	123.80 (9)
C5—C4—C3	119.26 (9)	C12—C13—N3	118.24 (9)
C5—C4—H4	120.4	C14—C13—N3	117.95 (8)
C3—C4—H4	120.4	C15—C14—C13	118.50 (9)
C4—C5—C6	120.36 (9)	C15—C14—H14	120.8
C4—C5—H5	119.8	C13—C14—H14	120.8
C6—C5—H5	119.8	C14—C15—C16	120.10 (9)
C5—C6—C1	121.10 (9)	C14—C15—H15	120.0
C5—C6—H6	119.4	C16—C15—H15	119.9
C1—C6—H6	119.4	N2—C16—C15	118.54 (9)
O2—C7—O1	124.46 (9)	N2—C16—C11	121.22 (9)
O2—C7—C1	122.25 (9)	C15—C16—C11	120.23 (9)
			
C6—C1—C2—C3	0.76 (15)	N2—C8—C9—C10	−1.0 (2)
C7—C1—C2—C3	179.34 (9)	C8—C9—C10—C11	1.07 (19)
C6—C1—C2—N1	−178.31 (9)	C9—C10—C11—C12	179.07 (11)
C7—C1—C2—N1	0.27 (15)	C9—C10—C11—C16	−0.01 (17)
O4—N1—C2—C3	90.22 (12)	C16—C11—C12—C13	1.04 (15)
O3—N1—C2—C3	−86.34 (12)	C10—C11—C12—C13	−178.04 (10)
O4—N1—C2—C1	−90.67 (12)	C11—C12—C13—C14	0.05 (16)
O3—N1—C2—C1	92.76 (12)	C11—C12—C13—N3	178.77 (9)
C1—C2—C3—C4	−0.19 (16)	O5—N3—C13—C12	4.41 (15)
N1—C2—C3—C4	178.92 (9)	O6—N3—C13—C12	−174.69 (9)
C1—C2—C3—Cl1	179.61 (8)	O5—N3—C13—C14	−176.79 (10)
N1—C2—C3—Cl1	−1.28 (14)	O6—N3—C13—C14	4.11 (14)
C2—C3—C4—C5	−0.26 (16)	C12—C13—C14—C15	−0.97 (16)
Cl1—C3—C4—C5	179.94 (8)	N3—C13—C14—C15	−179.70 (9)
C3—C4—C5—C6	0.12 (16)	C13—C14—C15—C16	0.76 (15)
C4—C5—C6—C1	0.48 (15)	C8—N2—C16—C15	−177.77 (10)
C2—C1—C6—C5	−0.90 (14)	C8—N2—C16—C11	1.38 (15)
C7—C1—C6—C5	−179.48 (9)	C14—C15—C16—N2	179.46 (9)
C2—C1—C7—O2	−1.69 (16)	C14—C15—C16—C11	0.30 (15)
C6—C1—C7—O2	176.84 (11)	C12—C11—C16—N2	179.64 (9)
C2—C1—C7—O1	179.50 (9)	C10—C11—C16—N2	−1.25 (15)
C6—C1—C7—O1	−1.96 (14)	C12—C11—C16—C15	−1.22 (15)
C16—N2—C8—C9	−0.26 (18)	C10—C11—C16—C15	177.89 (10)

### 3-Chloro-2-nitrobenzoicacid–6-nitroquinoline (1/1) (II) Hydrogen-bond geometry (Å, º)

**Table:**

*D*—H···*A*	*D*—H	H···*A*	*D*···*A*	*D*—H···*A*
O1—H1···N2	0.87 (3)	1.76 (3)	2.6310 (14)	176 (3)
C8—H8···O2	0.95	2.53	3.2082 (16)	128
C8—H8···O6^i^	0.95	2.41	3.2387 (15)	145
C14—H14···O4^ii^	0.95	2.52	3.3226 (16)	142
C12—H12···O5^iii^	0.95	2.37	3.2526 (16)	155

Symmetry codes: (i) *x*, *y*+1, *z*; (ii) *x*, *y*−1, *z*; (iii) −*x*+2, −*y*, −*z*+1.

### 8-Hydroxyquinoliniumb 3-chloro-2-nitrobenzoate (III) Crystal data

**Table:**

C_7_H_3_ClNO_4_·C_9_H_8_NO	*F*(000) = 712.00
*M**_r_* = 346.73	*D*_x_ = 1.561 Mg m^−^^3^
Monoclinic, *P*2_1_/*n*	Mo *K*α radiation, λ = 0.71075 Å
*a* = 7.3409 (5) Å	Cell parameters from 25520 reflections
*b* = 7.4689 (4) Å	θ = 3.0–30.0°
*c* = 27.0427 (14) Å	µ = 0.29 mm^−^^1^
β = 95.7158 (19)°	*T* = 190 K
*V* = 1475.33 (15) Å^3^	Block, pale yellow
*Z* = 4	0.45 × 0.30 × 0.26 mm

### 8-Hydroxyquinoliniumb 3-chloro-2-nitrobenzoate (III) Data collection

**Table:**

Rigaku R-AXIS RAPIDII diffractometer	3937 reflections with *I* > 2σ(*I*)
Detector resolution: 10.000 pixels mm^-1^	*R*_int_ = 0.019
ω scans	θ_max_ = 30.0°, θ_min_ = 3.0°
Absorption correction: numerical (*NUMABS*; Higashi, 1999)	*h* = −10→10
*T*_min_ = 0.844, *T*_max_ = 0.927	*k* = −10→10
29560 measured reflections	*l* = −37→37
4311 independent reflections	

### 8-Hydroxyquinoliniumb 3-chloro-2-nitrobenzoate (III) Refinement

**Table:**

Refinement on *F*^2^	Primary atom site location: structure-invariant direct methods
Least-squares matrix: full	Secondary atom site location: difference Fourier map
*R*[*F*^2^ > 2σ(*F*^2^)] = 0.033	Hydrogen site location: mixed
*wR*(*F*^2^) = 0.092	H atoms treated by a mixture of independent and constrained refinement
*S* = 1.06	*w* = 1/[σ^2^(*F*_o_^2^) + (0.0517*P*)^2^ + 0.422*P*] where *P* = (*F*_o_^2^ + 2*F*_c_^2^)/3
4311 reflections	(Δ/σ)_max_ = 0.001
225 parameters	Δρ_max_ = 0.39 e Å^−^^3^
0 restraints	Δρ_min_ = −0.29 e Å^−^^3^

### 8-Hydroxyquinoliniumb 3-chloro-2-nitrobenzoate (III) Special details

**Table:**

Geometry. All esds (except the esd in the dihedral angle between two l.s. planes) are estimated using the full covariance matrix. The cell esds are taken into account individually in the estimation of esds in distances, angles and torsion angles; correlations between esds in cell parameters are only used when they are defined by crystal symmetry. An approximate (isotropic) treatment of cell esds is used for estimating esds involving l.s. planes.

### 8-Hydroxyquinoliniumb 3-chloro-2-nitrobenzoate (III) Fractional atomic coordinates and isotropic or equivalent isotropic displacement parameters (Å^2^)

**Table:**

	*x*	*y*	*z*	*U*_iso_*/*U*_eq_	
Cl1	0.86076 (4)	0.71237 (3)	0.49827 (2)	0.03111 (8)	
O1	0.49893 (14)	0.57049 (11)	0.27915 (3)	0.0388 (2)	
O2	0.50879 (12)	0.37292 (10)	0.34157 (3)	0.03253 (18)	
O3	0.81650 (12)	0.34758 (10)	0.42238 (3)	0.03453 (19)	
O4	0.56116 (14)	0.41075 (12)	0.45203 (3)	0.0406 (2)	
O5	0.25608 (11)	0.64218 (10)	0.18109 (3)	0.03011 (17)	
N1	0.68835 (12)	0.44696 (11)	0.42827 (3)	0.02456 (17)	
N2	0.41002 (11)	0.32651 (12)	0.21056 (3)	0.02279 (16)	
C1	0.63355 (12)	0.66248 (12)	0.35750 (3)	0.02025 (17)	
C2	0.69708 (12)	0.62802 (12)	0.40684 (3)	0.01940 (16)	
C3	0.77981 (12)	0.76019 (13)	0.43755 (3)	0.02089 (17)	
C4	0.80144 (13)	0.93180 (13)	0.41926 (4)	0.02407 (18)	
H4	0.857461	1.022784	0.440109	0.029*	
C5	0.74011 (14)	0.96853 (13)	0.37014 (4)	0.02617 (19)	
H5	0.754706	1.085267	0.357150	0.031*	
C6	0.65741 (13)	0.83530 (13)	0.33982 (4)	0.02399 (18)	
H6	0.616164	0.862693	0.306263	0.029*	
C7	0.53938 (13)	0.52246 (13)	0.32319 (4)	0.02389 (18)	
C8	0.47845 (15)	0.17222 (14)	0.22785 (4)	0.0276 (2)	
H8	0.530731	0.164820	0.261364	0.033*	
C9	0.47520 (16)	0.02008 (14)	0.19773 (4)	0.0314 (2)	
H9	0.520396	−0.091226	0.210789	0.038*	
C10	0.40556 (15)	0.03461 (14)	0.14900 (4)	0.0292 (2)	
H10	0.404556	−0.067323	0.127964	0.035*	
C11	0.33516 (13)	0.19899 (13)	0.12955 (4)	0.02402 (19)	
C12	0.26151 (15)	0.22187 (16)	0.07953 (4)	0.0303 (2)	
H12	0.260234	0.124767	0.056813	0.036*	
C13	0.19225 (15)	0.38479 (17)	0.06420 (4)	0.0317 (2)	
H13	0.145274	0.400180	0.030447	0.038*	
C14	0.18881 (14)	0.53056 (16)	0.09724 (4)	0.0287 (2)	
H14	0.138468	0.641773	0.085598	0.034*	
C15	0.25798 (13)	0.51294 (13)	0.14634 (4)	0.02325 (18)	
C16	0.33572 (12)	0.34640 (13)	0.16246 (3)	0.02087 (17)	
H2	0.421 (2)	0.417 (2)	0.2315 (6)	0.044 (4)*	
H5O	0.173 (3)	0.722 (2)	0.1717 (7)	0.049 (5)*	

### 8-Hydroxyquinoliniumb 3-chloro-2-nitrobenzoate (III) Atomic displacement parameters (Å^2^)

**Table:**

	*U*^11^	*U*^22^	*U*^33^	*U*^12^	*U*^13^	*U*^23^
Cl1	0.04386 (16)	0.02719 (13)	0.02051 (12)	−0.00200 (9)	−0.00565 (9)	−0.00222 (8)
O1	0.0621 (6)	0.0278 (4)	0.0233 (4)	−0.0053 (4)	−0.0116 (3)	−0.0018 (3)
O2	0.0403 (4)	0.0219 (3)	0.0328 (4)	−0.0084 (3)	−0.0092 (3)	0.0002 (3)
O3	0.0389 (4)	0.0186 (3)	0.0436 (5)	0.0042 (3)	−0.0082 (3)	0.0010 (3)
O4	0.0546 (5)	0.0327 (4)	0.0368 (4)	−0.0104 (4)	0.0154 (4)	0.0047 (3)
O5	0.0389 (4)	0.0219 (3)	0.0282 (4)	0.0071 (3)	−0.0033 (3)	−0.0022 (3)
N1	0.0342 (4)	0.0172 (3)	0.0211 (4)	−0.0033 (3)	−0.0036 (3)	0.0004 (3)
N2	0.0267 (4)	0.0202 (4)	0.0214 (4)	0.0002 (3)	0.0019 (3)	−0.0013 (3)
C1	0.0217 (4)	0.0178 (4)	0.0206 (4)	0.0009 (3)	−0.0011 (3)	−0.0016 (3)
C2	0.0219 (4)	0.0152 (4)	0.0208 (4)	0.0006 (3)	0.0008 (3)	0.0002 (3)
C3	0.0228 (4)	0.0187 (4)	0.0206 (4)	0.0012 (3)	−0.0007 (3)	−0.0023 (3)
C4	0.0256 (4)	0.0165 (4)	0.0292 (5)	−0.0006 (3)	−0.0022 (3)	−0.0030 (3)
C5	0.0300 (5)	0.0161 (4)	0.0315 (5)	0.0002 (3)	−0.0013 (4)	0.0023 (3)
C6	0.0277 (4)	0.0194 (4)	0.0240 (4)	0.0016 (3)	−0.0016 (3)	0.0025 (3)
C7	0.0263 (4)	0.0205 (4)	0.0236 (4)	0.0007 (3)	−0.0040 (3)	−0.0032 (3)
C8	0.0318 (5)	0.0250 (5)	0.0259 (4)	0.0026 (4)	0.0025 (4)	0.0029 (4)
C9	0.0361 (5)	0.0219 (5)	0.0368 (5)	0.0044 (4)	0.0066 (4)	0.0014 (4)
C10	0.0321 (5)	0.0219 (4)	0.0348 (5)	−0.0013 (4)	0.0090 (4)	−0.0065 (4)
C11	0.0228 (4)	0.0249 (4)	0.0249 (4)	−0.0035 (3)	0.0053 (3)	−0.0040 (3)
C12	0.0300 (5)	0.0367 (6)	0.0246 (5)	−0.0062 (4)	0.0042 (4)	−0.0083 (4)
C13	0.0289 (5)	0.0450 (6)	0.0207 (4)	−0.0029 (4)	0.0002 (3)	−0.0003 (4)
C14	0.0272 (4)	0.0335 (5)	0.0251 (5)	0.0015 (4)	0.0012 (4)	0.0054 (4)
C15	0.0232 (4)	0.0229 (4)	0.0237 (4)	−0.0004 (3)	0.0024 (3)	0.0006 (3)
C16	0.0202 (4)	0.0212 (4)	0.0214 (4)	−0.0019 (3)	0.0030 (3)	−0.0010 (3)

### 8-Hydroxyquinoliniumb 3-chloro-2-nitrobenzoate (III) Geometric parameters (Å, º)

**Table:**

Cl1—C3	1.7268 (10)	C5—C6	1.3898 (14)
O1—C7	1.2511 (12)	C5—H5	0.9500
O2—C7	1.2519 (13)	C6—H6	0.9500
O3—N1	1.2211 (12)	C8—C9	1.3969 (15)
O4—N1	1.2154 (12)	C8—H8	0.9500
O5—C15	1.3483 (12)	C9—C10	1.3695 (16)
O5—H5O	0.872 (19)	C9—H9	0.9500
N1—C2	1.4751 (12)	C10—C11	1.4132 (15)
N2—C8	1.3236 (13)	C10—H10	0.9500
N2—C16	1.3676 (12)	C11—C16	1.4154 (13)
N2—H2	0.880 (16)	C11—C12	1.4161 (14)
C1—C2	1.3930 (12)	C12—C13	1.3671 (17)
C1—C6	1.3935 (13)	C12—H12	0.9500
C1—C7	1.5180 (13)	C13—C14	1.4103 (16)
C2—C3	1.3902 (12)	C13—H13	0.9500
C3—C4	1.3887 (13)	C14—C15	1.3798 (14)
C4—C5	1.3870 (14)	C14—H14	0.9500
C4—H4	0.9500	C15—C16	1.4189 (13)
			
C15—O5—H5O	109.9 (12)	N2—C8—C9	121.23 (10)
O4—N1—O3	125.17 (9)	N2—C8—H8	119.4
O4—N1—C2	118.51 (9)	C9—C8—H8	119.4
O3—N1—C2	116.22 (9)	C10—C9—C8	118.66 (10)
C8—N2—C16	122.14 (9)	C10—C9—H9	120.7
C8—N2—H2	115.9 (11)	C8—C9—H9	120.7
C16—N2—H2	121.9 (11)	C9—C10—C11	120.97 (9)
C2—C1—C6	117.27 (8)	C9—C10—H10	119.5
C2—C1—C7	123.14 (8)	C11—C10—H10	119.5
C6—C1—C7	119.58 (8)	C10—C11—C16	117.63 (9)
C3—C2—C1	121.72 (8)	C10—C11—C12	123.44 (9)
C3—C2—N1	116.73 (8)	C16—C11—C12	118.92 (9)
C1—C2—N1	121.48 (8)	C13—C12—C11	119.49 (10)
C4—C3—C2	120.08 (9)	C13—C12—H12	120.3
C4—C3—Cl1	119.26 (7)	C11—C12—H12	120.3
C2—C3—Cl1	120.64 (7)	C12—C13—C14	121.63 (10)
C5—C4—C3	119.08 (9)	C12—C13—H13	119.2
C5—C4—H4	120.5	C14—C13—H13	119.2
C3—C4—H4	120.5	C15—C14—C13	120.53 (10)
C4—C5—C6	120.29 (9)	C15—C14—H14	119.7
C4—C5—H5	119.9	C13—C14—H14	119.7
C6—C5—H5	119.9	O5—C15—C14	125.04 (9)
C5—C6—C1	121.56 (9)	O5—C15—C16	116.43 (8)
C5—C6—H6	119.2	C14—C15—C16	118.52 (9)
C1—C6—H6	119.2	N2—C16—C11	119.30 (9)
O1—C7—O2	126.78 (9)	N2—C16—C15	119.84 (8)
O1—C7—C1	115.77 (9)	C11—C16—C15	120.85 (9)
O2—C7—C1	117.44 (8)		
			
C6—C1—C2—C3	0.47 (14)	C16—N2—C8—C9	−1.11 (16)
C7—C1—C2—C3	−178.81 (9)	N2—C8—C9—C10	2.40 (16)
C6—C1—C2—N1	−176.32 (8)	C8—C9—C10—C11	−1.12 (16)
C7—C1—C2—N1	4.41 (14)	C9—C10—C11—C16	−1.33 (15)
O4—N1—C2—C3	85.64 (11)	C9—C10—C11—C12	−179.99 (10)
O3—N1—C2—C3	−91.03 (11)	C10—C11—C12—C13	178.41 (10)
O4—N1—C2—C1	−97.42 (11)	C16—C11—C12—C13	−0.23 (14)
O3—N1—C2—C1	85.91 (11)	C11—C12—C13—C14	−1.28 (16)
C1—C2—C3—C4	−0.17 (14)	C12—C13—C14—C15	0.76 (16)
N1—C2—C3—C4	176.76 (8)	C13—C14—C15—O5	−177.90 (10)
C1—C2—C3—Cl1	−178.87 (7)	C13—C14—C15—C16	1.27 (15)
N1—C2—C3—Cl1	−1.94 (12)	C8—N2—C16—C11	−1.46 (14)
C2—C3—C4—C5	−0.23 (14)	C8—N2—C16—C15	177.62 (9)
Cl1—C3—C4—C5	178.49 (8)	C10—C11—C16—N2	2.62 (13)
C3—C4—C5—C6	0.32 (15)	C12—C11—C16—N2	−178.66 (9)
C4—C5—C6—C1	−0.01 (15)	C10—C11—C16—C15	−176.45 (9)
C2—C1—C6—C5	−0.38 (14)	C12—C11—C16—C15	2.27 (14)
C7—C1—C6—C5	178.92 (9)	O5—C15—C16—N2	−2.60 (13)
C2—C1—C7—O1	−176.23 (9)	C14—C15—C16—N2	178.16 (9)
C6—C1—C7—O1	4.51 (14)	O5—C15—C16—C11	176.47 (8)
C2—C1—C7—O2	4.37 (14)	C14—C15—C16—C11	−2.77 (14)
C6—C1—C7—O2	−174.89 (9)		

### 8-Hydroxyquinoliniumb 3-chloro-2-nitrobenzoate (III) Hydrogen-bond geometry (Å, º)

**Table:**

*D*—H···*A*	*D*—H	H···*A*	*D*···*A*	*D*—H···*A*
N2—H2···O1	0.880 (16)	1.776 (16)	2.6355 (12)	164.7 (14)
O5—H5*O*···O2^i^	0.872 (19)	1.756 (19)	2.6247 (12)	173.2 (19)
C4—H4···O3^ii^	0.95	2.49	3.1082 (12)	123

Symmetry codes: (i) −*x*+1/2, *y*+1/2, −*z*+1/2; (ii) *x*, *y*+1, *z*.
